# Altered Methylation Profile of Lymphocytes Is Concordant with Perturbation of Lipids Metabolism and Inflammatory Response in Obesity

**DOI:** 10.1155/2016/8539057

**Published:** 2015-12-21

**Authors:** Mette J. Jacobsen, Caroline M. Junker Mentzel, Ann Sofie Olesen, Thierry Huby, Claus B. Jørgensen, Romain Barrès, Merete Fredholm, David Simar

**Affiliations:** ^1^Animal Genetics, Department of Veterinary Clinical and Animal Science, Faculty of Health and Medical Sciences, University of Copenhagen, 1870 Frederiksberg, Denmark; ^2^Sorbonne Universités, UPMC Univ Paris 06, INSERM UMR_S 1166, Integrative Biology of Atherosclerosis Team, 75013 Paris, France; ^3^Institute of Cardiometabolism and Nutrition (ICAN), Pitié-Salpêtrière Hospital, 75013 Paris, France; ^4^The Novo Nordisk Foundation Center for Basic Metabolic Research, Faculty of Health and Medical Sciences, University of Copenhagen, 2200 Copenhagen, Denmark; ^5^Inflammation and Infection Research, School of Medical Sciences, UNSW Australia, Sydney, NSW 2052, Australia

## Abstract

Obesity is associated with immunological perturbations that contribute to insulin resistance. Epigenetic mechanisms can control immune functions and have been linked to metabolic complications, although their contribution to insulin resistance still remains unclear. In this study, we investigated the link between metabolic dysfunction and immune alterations with the epigenetic signature in leukocytes in a porcine model of obesity. Global DNA methylation of circulating leukocytes, adipose tissue leukocyte trafficking, and macrophage polarisation were established by flow cytometry. Adipose tissue inflammation and metabolic function were further characterised by quantification of metabolites and expression levels of genes associated with obesity and inflammation. Here we show that obese pigs showed bigger visceral fat pads, higher levels of circulating LDL cholesterol, and impaired glucose tolerance. These changes coincided with impaired metabolism, sustained macrophages infiltration, and increased inflammation in the adipose tissue. Those immune alterations were linked to global DNA hypermethylation in both B-cells and T-cells. Our results provide novel insight into the possible contribution of immune cell epigenetics into the immunological disturbances observed in obesity. The dramatic changes in the transcriptomic and epigenetic signature of circulating lymphocytes reinforce the concept that epigenetic processes participate in the increased immune cell activation and impaired metabolic functions in obesity.

## 1. Introduction

Obesity is associated with a wide range of complications, such as insulin resistance, type 2 diabetes, fatty liver, cardiovascular diseases, and cancer [1–3]. Abnormal adipose tissue expansion leads to a chronic low-grade inflammatory state, due to increased recruitment and infiltration of immune cells into the tissue [4]. In particular, the number of classically activated or M1 adipose tissue macrophages (ATMs) is increased in obesity, and these cells are key contributors to the proinflammatory environment through the secretion of cytokines [5, 6]. Both T- and B-cells contribute to the initiation and maintenance of adipose tissue inflammation and are responsible for the recruitment of macrophages [7, 8]. Such proinflammatory environment is an important contributor to the development of insulin resistance and type 2 diabetes [9, 10].

Both genetic and environmental factors contribute to the development of obesity and associated diseases. The DNA methylome, a molecular mechanism mediating the interplay between genetic and environmental factors, influences metabolic functions by regulating gene expression in specific cell types [11, 12]. Recent studies have reported the existence of a specific epigenetic signature in peripheral blood mononuclear cells (PBMCs) in obese subjects [13] with obese individuals characterised by a hypermethylation and greater variation in global DNA methylation than lean subjects [14, 15]. In T-cells, B-cells, and macrophages, epigenetic regulations of genes involved in trafficking and polarised activation have been reported [16–18] and candidate gene approaches have identified epigenetic regulations of the* TNFα* and* Leptin* genes in obesity [19, 20]. Thus, the epigenetic signature of circulating and infiltrated immune cells could play a significant role in the inflammatory process observed in obesity.

Pigs share a plethora of similarities with humans in terms of diet, genetics, and metabolism and are thus pertinent animal models to study obesity [21, 22]. The significant similarity in the genome further supports the possibilities to translate the research findings into humans [22]. In particular genes regulating immunological functions show preservation of orthology of more than 80% between pigs and humans, compared to less than 10% between human and mice [23]. Here, we developed a polygenetic pig model designed for elucidating molecular components underlying obesity. Our pigs were bred under controlled conditions (housed in the same building under the same environmental conditions with unrestricted access to food and water) and were monitored intensively during their lifespan, and diseased pigs were excluded from the study. Thus, confounding environmental factors that could potentially influence their epigenetic profile were limited.

Here, we hypothesised that obesity-related changes in immune functions are linked to epigenetic mechanisms, leading to metabolic disorders. Using a novel porcine model of obesity, we aimed at investigating the link between epigenetic changes in immune cells and their impact on immune cell trafficking and functions, as well as lipid and glucose metabolism. We show that obesity is characterised by increased immune cell infiltration in the adipose tissue and increased expression level of critical genes involved in immune response and lipid metabolism. Obesity was associated with increased global DNA methylation of subpopulations of immune cells. We propose that this specific epigenetic signature could represent an early marker associated with immune cell recruitment and activation in obesity.

## 2. Material and Methods

### 2.1. Animals

Pigs from two highly divergent breeds, that is, Duroc and Göttingen minipigs, were bred to generate an F2 population. To ensure segregation of obesity traits in the F2 population, we used a breed that has been bred for leanness for decades (Duroc) and a breed prone to obesity (Göttingen minipigs) as previously described [24]. All F1 pigs were generated in the same direction and intercrossed to produce the F2 progeny. We selected 18 pigs from this F2 progeny with the most extreme obese and lean phenotypes. The specific characteristics used for the selection were body mass index (BMI), abdominal circumference, and amount of retroperitoneal fat. The sex distribution was 7 males/3 females in the obese group and 7 males/1 female in the lean group.

All pigs were housed in the same production farm, with 10–15 animals per pen and* ad libitum* access to standard production pig feed and water [24]. None of the animals included in this study received any antibiotics. Animal care, maintenance, and experimental work were conducted according to the “Animal Maintenance Act” (Act 432 dated 09/06/2004) and with the approval from the Danish Animal Experimentation Board (J.nr. 2007/561-1434). The pigs were slaughtered (9–13 months old, slaughtering weight: 67–160 kg) at a commercial slaughterhouse after overnight fasting. Weight, length, and abdominal circumference were measured prior to slaughter and tissue and blood samples were collected at slaughter. The animals were euthanized according to approved procedures under the supervision of a veterinarian (electronic stunning and bleeding). The following visceral fat samples were collected and weighed: retroperitoneal fat, greater omentum, and mesenteric fat, with the latter measured from an 8 cm diameter large section of the mesenteric fat in the triangle between ileum and cecum.

### 2.2. Oral Glucose Tolerance Test (OGTT)

Pigs were fasted for 24 h and challenged with 4 mL/kg of a 50% glucose solution mixed in food as previously described [25]. The pigs consumed the glucose mix within 30 minutes and blood glucose levels were measured with a Freestyle Mini glucometer (Hermedico, Brøndby, Denmark) using a drop of blood from the ear vein at fasting and every 30 minutes for three hours after the challenge.

### 2.3. Plasma Lipids

Plasma lipid profile was established using an Autoanalyzer (Konelab 20, Thermo Fisher Scientific, Waltham, MA, USA) and commercial kits from Roche Diagnostics for total cholesterol (Roche Diagnostics, Basel, Switzerland) and from Thermo Electron (Thermo Fisher Scientific) for triglycerides (TG) and HDL-cholesterol (HDL-C). Fasting plasma LDL-C was calculated using the Friedewald formula [26].

### 2.4. Cell Isolation

#### 2.4.1. Adipose Tissue Stromal Vascular Fraction (SVF)

The SVF containing preadipocytes and mononuclear cells was isolated from the retroperitoneal adipose tissue (approximately 15 g) from 12 animals (6 lean and 6 obese) as previously described [27] with a few modifications. Briefly, the adipose tissue was quickly removed from the animal after slaughter and rinsed in 37°C phosphate buffer saline (PBS), supplemented with 1% Penicillin/Streptomycin solution and 1% bovine serum albumin (BSA). The tissue was minced with scissors, and 20 mL of prewarmed (37°C) 0.2% collagenase solution in Hanks' Balanced Salt Solution and 1% BSA was subsequently added and then incubated for approximately 90 min in a 37°C water bath. The digested material was filtered through a 200 *μ*m sterile nylon filter and washed with Dulbecco's Modified Eagle's Medium (DMEM) with 10% FBS and 4.5 g/L glucose, and the mature adipocytes were allowed to float. The pellet containing the preadipocytes and mononuclear cells was filtered through a 40 *μ*m nylon filter, followed by centrifugation for 10 min at 400 ×g. The supernatant was removed and the pellet was washed twice in DMEM and subsequently preserved in aliquots of approximately 1 × 10^6^ cells per vial in DMEM (45% FBS and 10% DMSO) by gradually decreasing the temperature by 1°C/min to −80°C and then kept in liquid nitrogen until further analysis.

#### 2.4.2. Peripheral Blood Mononuclear Cells

The PBMCs were isolated from 2 × 6 mL of blood using Lymphoprep (Progen, Heidelberg, Germany) and washed in RMPI-1640 (25 mM HEPES). The purified PBMCs were resuspended in autologous plasma and preserved in aliquots of approximately 5 × 10^6^ cells per vial in 40% RPMI-1640 (10% DMSO) by gradually decreasing the temperature by 1°C/min to −80°C and then transferred to liquid nitrogen until further analysis.

### 2.5. Flow Cytometry

#### 2.5.1. Surface Staining of SVF Cells

SVF cells (1 × 10^6^) were quickly thawed at 37°C, washed once in RPMI-1640, resuspended in PBS (1% BSA), and incubated with the following antibodies: CD3*ε*-PerCp5.5, CD4-PE, and CD8*α*-Alexa Fluor 647 (all from BD, New Jersey, USA), CD21-APC and CD203a-DyLight405 (both from Novus Biologicals, Littleton, CO, USA), CD11R3-FITC (AbD serotec, Puchheim, Germany), CD45-DyLight680 (LSBio, Seattle, WA, USA), CD163-Alexa Fluor 350 and NKp46-PE (both from Bioss Antibodies, Woburn, MA, USA), for 20 min at RT in the dark, washed once with PBS (1% BSA) and fixed in 1% PFA for immediate analysis on a LSRFortessa flow cytometer (BD) at the Biological Resources Imaging Laboratory (Flow Cytometry Facility, UNSW Australia, Australia). T-cells were defined as CD45^+^CD3^+^. Within the T-cells, T-helper cells were defined as CD45^+^CD3^+^CD4^+^, T-cytotoxic cells as CD45^+^CD3^+^CD8^+^, and double positive T-cells as CD45^+^CD3^+^CD4^+^CD8^+^. Macrophages were defined as CD45^+^CD3^−^CD203A^+^, and the M1 and M2 macrophages were determined based on positivity for CD11R3 (CD45^+^CD3^−^CD203A^+^CD11R3^+^) and CD163, respectively (CD45^+^CD3^−^CD203A^+^CD163^+^). B-cells were CD45^+^CD3^−^CD203A^−^CD21^+^ whereas NK-cells were CD45^+^CD3^−^CD203A^−^NKp46^+^.

#### 2.5.2. Surface Staining of PBMCs

PBMCs (5 × 10^6^ cells) were quickly thawed at 37°C, washed once in RPMI-1640, and resuspended in PBS (1% BSA). They were then incubated with the following antibodies: CD3*ε*-PerCp5.5, CD4-PE, and CD8*α*-Alexa Fluor 647 or CD3*ε*-PerCp5.5, NKp46-PE, CD21-APC, and monocyte/granulocyte marker-PE (BD), for 20 min at room temperature. Cells were then washed twice with PBS (1% BSA) and fixed in 300 *μ*L PBS 1% PFA for immediate analysis using a FACSCalibur flow cytometer (BD). Lymphocytes and monocytes were defined based on size and granularity as well as the monocytes/granulocytes marker. T-cells, B-cells, and NK-cells were defined as described above, except for the omission of CD203A staining.

#### 2.5.3. DNA Methylation in PBMCs

Global DNA methylation in PBMCs was measured as previously described [14] with a few modifications. Briefly, PBMCs (10 × 10^6^ cells) were quickly thawed at 37°C, washed twice in RPMI-1640, resuspended in PBS (1% BSA), and incubated with CD4-PE and CD8*α*-Alexa Fluor 647 for 20 min at room temperature. Cells were then fixed in 2% PFA for 10 min at 37°C and permeabilised in 90% methanol for 30 min at −20°C. After three washes in PBS (1% BSA and 0.1% Tween-20), DNA was denaturated by adding 2 N HCl for 30 min (37°C) and cells were subsequently incubated in 0.1 M borate buffer for 5 min and washed three times again. PBMCs were finally resuspended in PBS (1% BSA, 0.1% Tween-20) and the following antibodies, CD3*ε*-PerCp5.5, CD21-APC (BD) and monocyte/granulocyte-PE, and 5-methylcytidine-DyLight488 (Novus Biologicals), were added for an incubation period of 20 min at room temperature in the dark. After two washes, cells were fixed in 1% PFA and immediately acquired on a FACSCalibur (BD).

### 2.6. Analysis of Flow Cytometry Data

All the FACS data were analysed using FlowJo software v10.0.7 (Ashland, OR, USA) and normalized using isotype controls. For the acquisition on the FACSCalibur, compensations were performed on single stained control samples before acquisition. Data collected on LSRFortessa was analysed with post-compensation on FlowJo using compensation beads from Beckman Coulter (Brea, CA, USA).

### 2.7. Total RNA Isolation and High-Throughput QPCR

Total RNA was purified from retroperitoneal adipose tissue from all 18 animals as previously described [28]. PBMCs (10 × 10^6^ cells) were quickly thawed at 37°C, washed once in RPMI-1640, and then resuspended in 1 mL of TriReagent (Molecular Research Center, Cincinnati, OH, USA). Total RNA was then purified [28] with the omission of the additional centrifugation step to remove lipids. RNA concentration was estimated using a NanoDrop 1000 (Thermo Fisher Scientific) and the quality was assessed on an Experion system (Bio-Rad, Hercules, CA, USA), with all RQI values between 7.8 and 9.9. cDNA synthesis was performed on 400 ng in duplicate using Improm-IITM reverse transcriptase (Promega, Madison, WI, USA) and a 3 : 1 mixture of random hexamers/OligodT, according to the manufacturer's recommendations. cDNA was diluted 1/16 prior to the QPCR.

QPCR was performed on the Biomark HD 96.96 IFC chip (Fluidigm Corporation, San Francisco, CA, USA) according to the manufacturer's protocol and data collected using the associated software. Raw Cq values were subsequently transferred to Genex5 Pro (MultiD, Göteborg, Sweden), and the relative expression levels of the tested genes were normalised to* RPL4*, as this gene showed the highest stability in both adipose tissue and PBMC samples. Technical replicates were averaged, and data was log transformed to achieve normal distribution. The genes, primer sequences, and normalised log⁡(2) transformed fold changes (FC) are all listed in Supplementary Table 1 in the Supplementary Material available online at http://dx.doi.org/10.1155/2016/8539057.

QPCR of* SSP1*,* SREBF1*,* IL4*,* ADIPOR1*, and* RPL4* was performed on Mx3005P (Agilent, Santa Clara, CA, USA) using QuantiFast SYBR Green PCR Master Mix (Qiagen, Hilden, Germany) according to supplier's instruction and analysed in Genex5 Pro as described above.

### 2.8. Statistical Analysis

Values are expressed as mean ± SD and statistical analysis was performed on SPSS Statistics for Mac (Version 22, IBM, USA). Normality of the distribution was tested using the D'Agostino skewness test and the Anscombe-Glynn kurtosis test. In case of nonnormal distribution, data was log transformed to achieve normal distribution. Differences between the two groups were tested using unpaired Student's *t*-test. Data from the OGTT was analysed by ANOVA for repeated measures. Statistical significance was set at *p* < 0.05.

## 3. Results

### 3.1. Animal Characteristics

**Figure 1:**
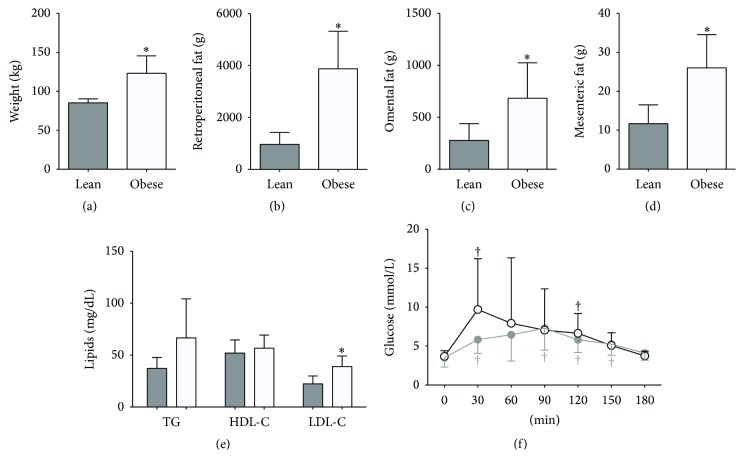
Anthropometric and metabolic characteristics in the lean group of animals (*n* = 8, grey columns and line) and in the obese group of animals (*n* = 10, open columns and a black line). The animals were weighted before slaughter (a), and after slaughter the weight of the retroperitoneal fat (b), omental fat (c), and mesenteric fat (d) was measured. Triglycerides (TG), high-density lipoprotein (HDL-C), and low-density lipoprotein (LDL-C) were measured from plasma collected after an overnight fast (e). Oral glucose tolerance test (OGTT) was performed only on males (7 in each group) after an overnight fast, and blood glucose was measured every 30 min for 3 hours (f). Data are mean ± SD. ^*∗*^ *p* < 0.05 compared to lean. ^†^ *p* < 0.05 compared to baseline.

The animals used in this study were selected from a population designed to exhibit large variation in obesity traits. The obese group of pigs had significantly higher body weight and increased mass in the different fat pads (Figures 1(a)–1(d), *p* < 0.05), whereas both age (10.4 ± 1.6* versus *9.3 ± 0.7 months) and length (86.4 ± 5.2* versus *90.4 ± 8.5 cm) did not differ significantly. The measured fat pads, retroperitoneal, omental, and mesenteric, are all part of the abdominal visceral fat depots, in which adipocyte sizes and numbers have been highly correlated with metabolic diseases. Metabolic perturbations were clearly identified in the obese group, as circulating LDL-C levels were significantly higher (Figure 1(e)). Given that previous reports showed a sex-specific blood glucose variation in obesity [25], we only included male pigs in the analysis of the OGTT. We found no difference in fasting blood glucose but a trend towards impaired glucose clearance in the obese as compared to the lean animals (Figure 1(f)). Thus, our data supports that our model successfully recapitulated the obese phenotype typically reported in humans.

### 3.2. Altered Immune Cells Distribution in Both Adipose Tissue and Circulating Leukocytes

**Figure 2:**
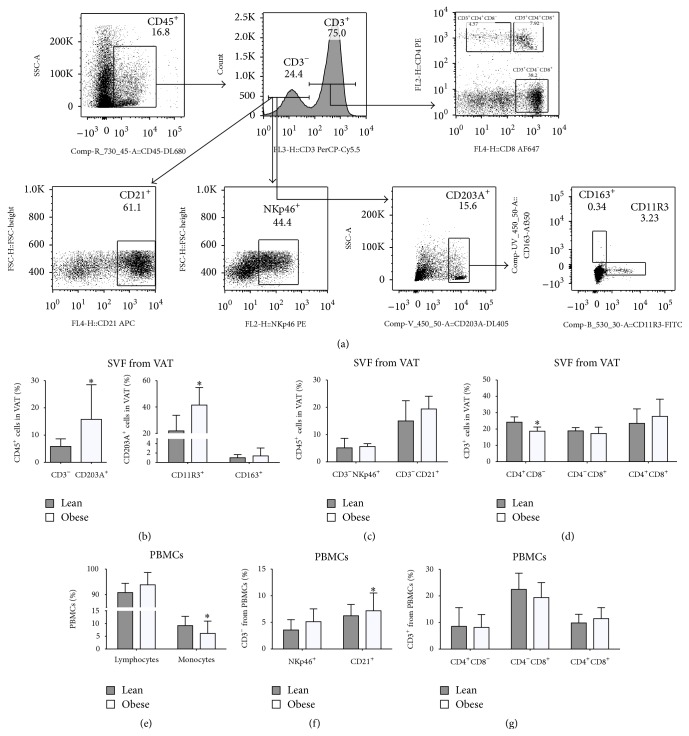
Phenotypic characterisation by flow cytometry of the adipose SVF cells and PBMCs in lean and obese pigs. (a) Gating strategy of the porcine adipose SVF cells from an obese pig. T-cells were defined as CD45^+^CD3^+^. Within the T-cells, CD4^+^, CD8^+^, and CD4^+^CD8^+^ were characterised based on positivity for CD4 and CD8. Macrophages were defined as CD45^+^CD3^−^CD203A^+^, and the M1 and M2 macrophages were determined based on positivity for CD11R3 and CD163, respectively. B-cells were CD45^+^CD3^−^CD203A^−^ and CD21^+^ whereas NK-cells were CD45^+^CD3^−^CD203A^−^ and NKp46^+^. (b–d) Infiltrating leukocyte populations in the adipose tissue within the CD45^+^ population from lean (grey columns) and obese (white columns) pigs. (b) Frequency of the total macrophages (CD203A^+^), classically activated M1 macrophages (CD203^+^CD11R3^+^), and alternatively activated M2 macrophages (CD203^+^CD163^+^). (c) Frequency of natural killer cells (NKp46^+^) and B-cells (CD21^+^). (d) Frequency of the T-helper (CD3^+^CD4^+^), T-cytotoxic (CD3^+^CD8^+^), and double positive T-cells (CD3^+^CD4^+^CD8^+^) within the T-cells. (e–g) PBMCs subpopulation frequencies. (e) Lymphocytes and monocytes. (f) Natural killer cells (CD3^−^NKp46^+^) and B-cells (CD3^−^CD21^+^) populations frequency. (g) Frequency of the T-helper (CD3^+^CD4^+^), T-cytotoxic (CD3^+^CD8^+^), and double positive T-cells (CD3^+^CD4^+^CD8^+^) within the T-cells. Data are mean ± SD. ^*∗*^ *p* < 0.05.

After extraction of the SVF from the retroperitoneal fat pads, we isolated leukocytes and phenotyped them using high-resolution 14-colour flow cytometry. The comparison of lean with obese pigs did not reveal any difference in the frequency of infiltrated T-cells, although the percentage of T-helper cells was decreased in obese animals (Figure 2(d)). The frequency of mature ATMs was increased in the obese group (Figure 2(b)) and was further associated with an increased frequency of M1 macrophages (Figure 2(b)), whereas no difference was observed for the frequency of M2 macrophages between the two groups (Figure 2(b)).

To determine if the proinflammatory phenotype we observed in the adipose tissue was linked to systemic immune changes, we investigated the cell type distribution in circulating PBMCs. Obese animals showed a higher percentage of B-cells (Figure 2(f)) and a lower frequency of monocytes (Figure 2(e)).

The increased macrophage infiltration we detected and, in particular, the proinflammatory macrophages, in conjunction with the decreased number of peripheral monocytes, suggests that an active recruitment of monocytes/macrophages occurs in the adipose tissue of obese animals.

### 3.3. Differential Gene Expression in Adipose Tissue and Circulating Leukocytes from Obese Pigs

**Figure 3:**
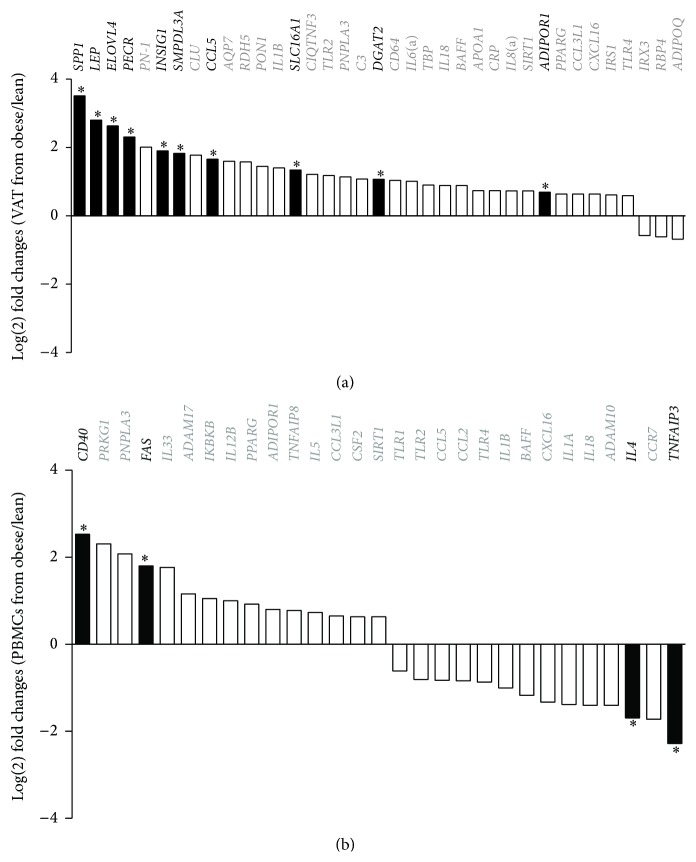
Expression profile of the genes that showed a log⁡(2) fold change ratio higher than 1.5. The log⁡(2) difference between lean and obese pigs in the retroperitoneal adipose tissue (a) and the PBMCs (b). +log⁡(2) FC: upregulated in obese pigs; −log⁡(2) FC: downregulated in obese pigs. Gene names and columns in black: significant differential expression. ^*∗*^ *p* < 0.05.

To investigate gene expression variation in obesity, we profiled RNA expression in both retroperitoneal adipose tissue and PBMCs from lean and obese pigs. In particular, we focused on adipokines, cytokines, chemokines, and genes known to be actively involved in obesity and inflammation. Of the 72 genes investigated, 60 were specific to the adipose tissue and 50 to the PBMCs. Sixty-six of these revealed a log⁡(2) fold change ratio of more than ±1.5 between the lean and obese animals (Figures 3(a) and 3(b)).

In adipose tissue, 37 genes were differentially expressed between the two groups and 10 of these were significantly upregulated in obese animals (Figure 3(a), black columns). Notably,* SPP1* (*Osteopontin*) and* CCL5* (*RANTES*), two cytokines highly expressed in inflammatory states, were overexpressed in the adipose tissue of the obese group. In addition,* LEP* (*Leptin*), a hormone involved in appetite regulation possessing proinflammatory properties, was increased in obese pigs. Genes controlling lipid metabolism were also affected:* INSIG1* (*insulin induced gene 1*), a regulator of cholesterol homeostasis,* ELOVL4* (*ELOVL fatty acid elongase 4*) and* PECR* (*peroxisomal trans-2-enoyl-CoA reductase*), both involved in fatty acid metabolism, and* DGAT2* (*diacylglycerol O-acyltransferase homolog 2*), which is involved in synthesis and storage of intercellular triglycerides, were upregulated in obese pigs.* SMPDL3A* (*sphingomyelin phosphodiesterase, acid-like 3A*), involved in cholesterol loading in macrophages [29], and* SLC16A1* (*solute carrier family 16*), a monocarboxylates transporter, were upregulated in obese pigs.

In PBMCs, a total of 29 genes showed more than 1.5log⁡(2) fold difference between the two groups, but only four genes were statistically significantly up- or downregulated (Figure 3(b), black columns).* CD40* and* FAS* (*TNF receptor superfamily members 5 and 6*) were both upregulated in the obese pigs. Both genes are essential for the initiation and progression of inflammation [30, 31].* TNFAIP3* (*tumor necrosis factor, alpha-induced protein 3*) and* IL4* (*interleukin 4*), both playing a regulatory role in the inflammatory cascade, were downregulated in the obese animals.

Collectively, our gene expression analyses show dysregulation of several key genes in both the inflammatory pathway and fatty acid metabolism, supporting a link between increased adiposity, impaired lipid metabolism, and activated immune response in the adipose tissue.

### 3.4. Global DNA Methylation Is Altered in Circulating Leukocytes

**Figure 4:**
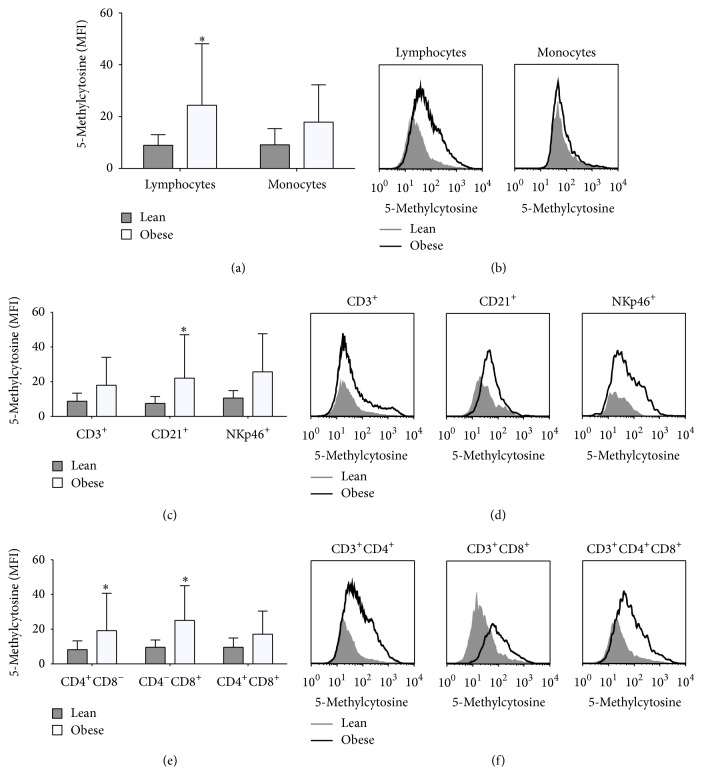
Global DNA methylation in PBMCs subpopulations from lean (grey columns and histograms) and obese pigs (open columns and histograms). (a) Representative quantification of 5-methylcytosine levels in lymphocytes and monocytes. (b) Quantification of 5-methylcytosine levels (MFI) in lymphocytes and monocytes. (c) Representative quantification of 5-methylcytosine levels in T-cells (CD3^+^), B-cells (CD21^+^), and natural killer cells (NKp46^+^). (d) Quantification of 5-methylcytosine levels (MFI) in T-cells, B-cells, and natural killer cells. (e) Representative quantification of 5-methylcytosine levels in T-cells subpopulations: T-helper (CD3^+^CD4^+^), T-cytotoxic (CD3^+^CD8^+^), and double positive T-cells (CD3^+^CD4^+^CD8^+^). (f) Quantification of 5-methylcytosine levels (MFI) in T-helper, T-cytotoxic, and double positive T-cells. Data are mean ± SD. ^*∗*^ *p* < 0.05. MFI: median fluorescence intensity.

An altered epigenetic signature in lymphocytes and PBMCs has previously been reported in obesity and metabolic disease [13–15, 32]. Epigenetic modifications in immune cells could be a mechanism by which the immune system is deregulated in metabolic disorders. We measured global DNA methylation levels in circulating PBMCs in a cell type specific manner (T-helper, T-cytotoxic, double positive T-cells, natural killer cells and B-cells) using FACS as previously reported [14]. In obese animals, we found higher levels of global DNA methylation in lymphocytes but not in monocytes (Figures 4(a) and 4(b)). The analysis of the individual lymphocyte subpopulations showed increased global DNA methylation in B-cells (Figures 4(c) and 4(d)), T-helper cells and T-cytotoxic cells (Figures 4(e) and 4(f)), suggesting that obesity is associated with altered epigenetic signature in specific subpopulations of circulating lymphocytes.

## 4. Discussion

This study aimed at investigating the link between obesity and altered trafficking and polarisation of immune cells in the adipose tissue and their potential association with epigenetic changes in a pig model of obesity. In obese pigs, impaired glucose tolerance and an altered lipid profile were associated with increased immune cell infiltration in the visceral adipose tissue and immune activation. These changes were further linked to an altered transcriptomic and epigenetic signature of circulating lymphocytes, which reinforces the concept that epigenetic processes participate in the increased immune cell activation and impaired metabolic functions in obesity.

To our knowledge, our study is the first of its kind to exploit differences in the development of obesity in F2 pigs produced by breeds that are highly divergent with respect to obesity, thus ensuring that the obese phenotype is predominantly genetically determined.

Pigs represent a useful model for obesity research, although the majority of pig studies have focused on either different diets or breed differences [25, 33–35]. It is noteworthy that our obese animals when compared to the lean ones had higher total body weight, visceral fat mass, and altered lipid profile, as well as impaired glucose tolerance. These results are consistent with previous reports investigating the effect of high fat diet (HFD) in pigs [25, 33, 36].

Altered infiltration of immune cells in the adipose tissue of obese pigs has previously been reported in the Ossabaw pig breed [35]. In this model, the frequency of CD203A^+^ macrophages was reduced in obese animals receiving a HFD for 30 weeks. In contrast, we report an increased infiltration of macrophages in obese animals, a finding generally observed in human and murine studies [6, 37, 38]. Particularly, the frequency of proinflammatory M1 macrophages was higher in the adipose tissue from obese pigs, supporting the validity of our model in recapitulating the specific immune environment in adipose tissue previously reported in human and murine obesity [5, 6].

The frequency of T-helper cells was significantly decreased in the obese pigs. In human and murine obesity studies, conflicting results have been reported concerning the frequency of this subpopulation of T-cells [8, 39–41]. Deiuliis et al. (2011) have reported a decrease of total CD4^+^ T-cells in obese humans, with a concomitant increase in activated T-effector cells. We did not investigate the specific frequency of the T-helper subpopulations here (effectors* versus* regulatory), which could have provided additional information, since both T-effector and T-regulatory cells (Tregs) seem to play critical roles in insulin resistance and diabetes [40, 42].

The precise sequence of inflammatory cytokine secretion and immune cell infiltration in the progression of adipose tissue inflammation is still not fully understood. The most recent studies have suggested that overnutrition and adipose tissue expansion cause the adipocytes to secrete various chemokines and cytokines, which in turn mediate the recruitment and the activation of immune cells to the adipose tissue. These recruited immune cells then initiate a second wave of inflammatory chemokine/cytokine production [9]. In our obese pigs, we found an increase of* CCL5* in the adipose tissue, and* CCL3L1* and* CXCL16* also trended to be upregulated. This could support increased trafficking of immune cell, as the upregulation of chemokines is implicated in the overall recruitment of leukocytes [43]. The upregulation of* Leptin* in obese pigs could also have contributed to the increase in immune cells trafficking to the adipose tissue since it has been shown to stimulate innate immune response by increasing chemotaxis and enhancing the secretion of proinflammatory cytokines [44]. Similarly, the upregulation of* SPP1* underpins an increased infiltration of immune cells and the resulting inflammatory process, as this gene is highly induced during inflammation in obesity [45]. A further evidence for immune activation is that interleukins* IL1B*,* IL6*,* IL8*, and* IL18* and the Toll-like receptors* TLR2* and* TLR4* also tended to be upregulated in the obese animals.

The decreased frequency of circulating monocytes in obese animals could also suggest that monocytes are recruited to the adipose tissue of obese animals. This is consistent with previous findings in high fat-fed mice, where a significant decrease of the peripheral monocyte population was observed, concomitant with an increased macrophage infiltration in adipose tissue [46]. We also found an increased number of B-cells in the blood stream of the obese pigs. This could be consistent with systemic immune activation as previously described [7]. Upregulation of* CD40* and* FAS* in PBMCs from obese pigs might reflect the increased frequency of circulating B-cells. Both CD40 and FAS have recently been shown to be increased in plasma from obese subjects and are highly expressed by B-cells [31, 47]. It can be speculated that the upregulation of these two proteins could be an indication of a proinflammatory environment in obese pigs, as both genes are associated with the regulation of immune response [47, 48]. In contrast,* TNFAIP3*, one of the key inhibitors of the nuclear factor-kB family, which plays a critical role in the regulation of inflammation, was downregulated in the obese group.* In vivo* gene targeting studies have established its importance in the regulation of inflammation in myeloid cells, B-cells, and macrophages that lack* TNFAIP3* [49, 50]. Similarly, the anti-inflammatory cytokine* IL4* was also downregulated in obese pigs, contributing to the proinflammatory environment and increased lipid accumulation [51].

The pigs used in this study were born at the same time of the year and housed in the same building under the same environmental conditions with free access to food and water, and none of them suffered from diseases that needed antibiotics/treatments during their lifespan. This very stringent and controlled environment suggests that the differences in epigenetic profile we report in these pigs are most likely to reflect variations due to obesity. Epigenetic regulation of immune cells has previously been associated with changes in cytokine production and could thereby influence the inflammatory environment associated with obesity [52]. The global DNA methylation profile of circulating immune cells was therefore established using flow cytometry allowing us to detect differences in specific subpopulations of PBMCs. We observed a global DNA hypermethylation in B-cells and T-helper and T-cytotoxic cells, consistent with our recent findings in B-cells from obese individuals [14]. In this previous study, hypermethylation in B- and NK-cells was associated with the severity of insulin resistance. We failed to observe such relation in our animals, which could be explained by the fact that these pigs had not yet developed a severe level of insulin resistance. It is noteworthy that we observed an increased global DNA methylation in T-helper, T-cytotoxic, and B-cells, since these cells not only have been linked to the development of obesity but also have been suggested to be part of the initiation and maintenance of inflammation in adipose tissue, contributing to the recruitment of macrophages and the development of insulin resistance [7, 8, 41, 53].

In summary, we have established a porcine model of obesity, which displays numerous characteristics of the human metabolic syndrome. We show that obese pigs are characterised by early defects in glucose metabolism and increased recruitment of monocytes/macrophages to the adipose tissue and exhibit a proinflammatory environment in both adipose tissue and peripheral blood. The altered epigenetic profile we detect in lymphocytes is likely to contribute to the proinflammatory environment and could therefore represent an early marker of immune cell recruitment and activation in obesity.

## Supplementary Material

Supplementary table 1: Gene information, primer sequences and normalized log(2) transformed fold changes (FC) for the High-Throughput QPCR.

## References

[1] Pedersen S. D. (2013). Metabolic complications of obesity. *Best Practice & Research: Clinical Endocrinology & Metabolism*.

[2] Koppe S. W. P. (2014). Obesity and the liver: nonalcoholic fatty liver disease. *Translational Research*.

[3] Davoodi S. H., Malek-Shahabi T., Malekshahi-Moghadam A., Shahbazi R., Esmaeili S. (2013). Obesity as an important risk factor for certain types of cancer. *Iranian Journal of Cancer Prevention*.

[4] Weisberg S. P., McCann D., Desai M., Rosenbaum M., Leibel R. L., Ferrante A. W. (2003). Obesity is associated with macrophage accumulation in adipose tissue. *The Journal of Clinical Investigation*.

[5] Aron-Wisnewsky J., Tordjman J., Poitou C. (2009). Human adipose tissue macrophages: M1 and M2 cell surface markers in subcutaneous and omental depots and after weight loss. *The Journal of Clinical Endocrinology & Metabolism*.

[6] Oh D. Y., Morinaga H., Talukdar S., Bae E. J., Olefsky J. M. (2012). Increased macrophage migration into adipose tissue in obese mice. *Diabetes*.

[7] DeFuria J., Belkina A. C., Jagannathan-Bogdan M. (2013). B cells promote inflammation in obesity and type 2 diabetes through regulation of T-cell function and an inflammatory cytokine profile. *Proceedings of the National Academy of Sciences of the United States of America*.

[8] Nishimura S., Manabe I., Nagasaki M. (2009). CD8^+^ effector T cells contribute to macrophage recruitment and adipose tissue inflammation in obesity. *Nature Medicine*.

[9] Gregor M. F., Hotamisligil G. S. (2011). Inflammatory mechanisms in obesity. *Annual Review of Immunology*.

[10] Sell H., Eckel J. (2009). Chemotactic cytokines, obesity and type 2 diabetes: in vivo and in vitro evidence for a possible causal correlation?. *Proceedings of the Nutrition Society*.

[11] de Mello V. D. F., Pulkkinen L., Lalli M., Kolehmainen M., Pihlajamäki J., Uusitupa M. (2014). DNA methylation in obesity and type 2 diabetes. *Annals of Medicine*.

[12] van Dijk S. J., Molloy P. L., Varinli H. (2015). Epigenetics and human obesity. *International Journal of Obesity*.

[13] Wang X., Zhu H., Snieder H. (2010). Obesity related methylation changes in DNA of peripheral blood leukocytes. *BMC Medicine*.

[14] Simar D., Versteyhe S., Donkin I. (2014). DNA methylation is altered in B and NK lymphocytes in obese and type 2 diabetic human. *Metabolism: Clinical and Experimental*.

[15] Xu X., Su S., Barnes V. A. (2013). A genome-wide methylation study on obesity: differential variability and differential methylation. *Epigenetics*.

[16] Janson P. C. J., Linton L. B., Bergman E. A. (2011). Profiling of CD4^+^ T cells with epigenetic immune lineage analysis. *Journal of Immunology*.

[17] Yang X., Wang X., Liu D., Yu L., Xue B., Shi H. (2014). Epigenetic regulation of macrophage polarization by DNA methyltransferase 3b. *Molecular Endocrinology*.

[18] Maier H., Ostraat R., Gao H. (2004). Early B cell factor cooperates with Runx1 and mediates epigenetic changes associated with mb-1 transcription. *Nature Immunology*.

[19] Hermsdorff H. H., Mansego M. L., Campión J., Milagro F. I., Zulet M. A., Martínez J. A. (2013). TNF-alpha promoter methylation in peripheral white blood cells: relationship with circulating TNFalpha, truncal fat and n-6 PUFA intake in young women. *Cytokine*.

[20] García-Cardona M. C., Huang F., García-Vivas J. M. (2014). DNA methylation of leptin and adiponectin promoters in children is reduced by the combined presence of obesity and insulin resistance. *International Journal of Obesity*.

[21] Spurlock M. E., Gabler N. K. (2008). The development of porcine models of obesity and the metabolic syndrome. *The Journal of Nutrition*.

[22] Groenen M. A., Archibald A. L., Uenishi H. (2012). Analyses of pig genomes provide insight into porcine demography and evolution. *Nature*.

[23] Dawson H. D., Dayan A., Hastings K. (2011). Comparative assessment of the pig, mouse, and human genomes; a structural and functional analysis of genes involved in immunity. *The Minipig in Biomedical Research P A McAnulty*.

[24] Kogelman L. J. A., Kadarmideen H. N., Mark T. (2013). An F2 pig resource population as a model for genetic studies of obesity and obesity-related diseases in humans: design and genetic parameters. *Frontiers in Genetics*.

[25] Christoffersen B., Golozoubova V., Pacini G., Svendsen O., Raun K. (2013). The young Göttingen minipig as a model of childhood and adolescent obesity: influence of diet and gender. *Obesity*.

[26] Friedewald W. T., Levy R. I., Fredrickson D. S. (1972). Estimation of the concentration of low-density lipoprotein cholesterol in plasma, without use of the preparative ultracentrifuge. *Clinical Chemistry*.

[27] Decaunes P., Estève D., Zakaroff-Girard A., Sengenès C., Galitzky J., Bouloumié A. (2011). Adipose-derived stromal cells: cytokine expression and immune cell contaminants. *Methods in Molecular Biology*.

[28] Cirera S. (2013). Highly efficient method for isolation of total RNA from adipose tissue. *BMC Research Notes*.

[29] Traini M., Quinn C. M., Sandoval C. (2014). Sphingomyelin phosphodiesterase acid-like 3A (SMPDL3A) is a novel nucleotide phosphodiesterase regulated by cholesterol in human macrophages. *The Journal of Biological Chemistry*.

[30] Foy T. M., Aruffo A., Bajorath J., Buhlmann J. E., Noelle R. J. (1996). Immune regulation by CD40 and its ligand GP39. *Annual Review of Immunology*.

[31] Wueest S., Mueller R., Blüher M. (2014). Fas (CD95) expression in myeloid cells promotes obesity-induced muscle insulin resistance. *EMBO Molecular Medicine*.

[32] Huang Y.-T., Maccani J. Z. J., Hawley N. L., Wing R. R., Kelsey K. T., McCaffery J. M. (2015). Epigenetic patterns in successful weight loss maintainers: a pilot study. *International Journal of Obesity*.

[33] Newell-Fugate A. E., Taibl J. N., Clark S. G., Alloosh M., Sturek M., Krisher R. L. (2014). Effects of diet-induced obesity on metabolic parameters and reproductive function in female ossabaw minipigs. *Comparative Medicine*.

[34] Cirera S., Jensen M. S., Elbrønd V. S. (2014). Expression studies of six human obesity-related genes in seven tissues from divergent pig breeds. *Animal Genetics*.

[35] Faris R. J., Boddicker R. L., Walker-Daniels J., Li J., Jones D. E., Spurlock M. E. (2012). Inflammation in response to n3 fatty acids in a porcine obesity model. *Comparative Medicine*.

[36] Pawar A. S., Zhu X.-Y., Eirin A. (2015). Adipose tissue remodeling in a novel domestic porcine model of diet-induced obesity. *Obesity*.

[37] Amano S. U., Cohen J. L., Vangala P. (2014). Local proliferation of macrophages contributes to obesity-associated adipose tissue inflammation. *Cell Metabolism*.

[38] Patel P. S., Buras E. D., Balasubramanyam A. (2013). The role of the immune system in obesity and insulin resistance. *Journal of Obesity*.

[39] Kintscher U., Hartge M., Hess K. (2008). T-lymphocyte infiltration in visceral adipose tissue: a primary event in adipose tissue inflammation and the development of obesity-mediated insulin resistance. *Arteriosclerosis, Thrombosis, and Vascular Biology*.

[40] Deiuliis J., Shah Z., Shah N. (2011). Visceral adipose inflammation in obesity is associated with critical alterations in tregulatory cell numbers. *PLoS ONE*.

[41] Winer S., Chan Y., Paltser G. (2009). Normalization of obesity-associated insulin resistance through immunotherapy. *Nature Medicine*.

[42] Cipolletta D., Feuerer M., Li A. (2012). PPAR-*γ* is a major driver of the accumulation and phenotype of adipose tissue T reg cells. *Nature*.

[43] Yao L., Herlea-Pana O., Heuser-Baker J., Chen Y., Barlic-Dicen J. (2014). Roles of the chemokine system in development of obesity, insulin resistance, and cardiovascular disease. *Journal of Immunology Research*.

[44] Carbone F., La Rocca C., Matarese G. (2012). Immunological functions of leptin and adiponectin. *Biochimie*.

[45] Lund S. A., Giachelli C. M., Scatena M. (2009). The role of osteopontin in inflammatory processes. *Journal of Cell Communication and Signaling*.

[46] Ingvorsen C., Thysen A. H., Fernandez-Twinn D. (2014). Effects of pregnancy on obesity-induced inflammation in a mouse model of fetal programming. *International Journal of Obesity*.

[47] Wolf D., Jehle F., Michel N. A. (2014). Coinhibitory suppression of T cell activation by CD40 protects against obesity and adipose tissue inflammation in mice. *Circulation*.

[48] Dhein J., Walczak H., Baumler C., Debatin K.-M., Krammer P. H. (1995). Autocrine T-cell suicide mediated by APO-1/(Fas/CD95). *Nature*.

[49] Catrysse L., Vereecke L., Beyaert R., van Loo G. (2014). A20 in inflammation and autoimmunity. *Trends in Immunology*.

[50] Matmati M., Jacques P., Maelfait J. (2011). A20 (TNFAIP3) deficiency in myeloid cells triggers erosive polyarthritis resembling rheumatoid arthritis. *Nature Genetics*.

[51] Tsao C.-H., Shiau M.-Y., Chuang P.-H., Chang Y.-H., Hwang J. (2014). Interleukin-4 regulates lipid metabolism by inhibiting adipogenesis and promoting lipolysis. *Journal of Lipid Research*.

[52] Lawson B. R., Eleftheriadis T., Tardif V. (2012). Transmethylation in immunity and autoimmunity. *Clinical Immunology*.

[53] Winer D. A., Winer S., Shen L. (2011). B cells promote insulin resistance through modulation of T cells and production of pathogenic IgG antibodies. *Nature Medicine*.

